# Assessment of Prevalence and Determinants of Occupational Exposure to HIV Infection among Healthcare Workers in Selected Health Institutions in Debre Berhan Town, North Shoa Zone, Amhara Region, Ethiopia, 2014

**DOI:** 10.1155/2014/731848

**Published:** 2014-11-13

**Authors:** Filmawit Aynalem Tesfay, Tesfa Dejenie Habtewold

**Affiliations:** Department of Nursing, College of Health Science, Debre Berhan University, Debre Berhan, Ethiopia

## Abstract

*Introduction*. Health care workers are exposed to different kinds of occupational hazards due to their day to day activities. The most common occupational exposure like body fluids is a potential risk of transmission of blood-borne infection like human immunodeficiency virus.* Objective.* To assess the prevalence and determinants of occupational exposure to human immunodeficiency virus infection.* Methods and Materials*. A descriptive cross-sectional institution based study was conducted in selected four health institutions in Debre Berhan town. Quantitative and qualitative data were collected using semistructured interviewer administered questionnaire. The frequency distribution of dependent and independent variables was worked out and presented using frequency table, graph, and chart.* Result*. The overall prevalence of occupational exposure of the health care workers was found to be 88.6% (*n* = 187) in the past 12 months. Contact to potentially infectious body fluids accounts for the largest proportion (56.7%) followed by needle stick injury (31.5%) and glove breakage (28.8%).* Conclusion*. In this study majority (88.6%) of the health care workers had a risky occupational hazard that exposed them to human immunodeficiency virus infection during the past 12 months. The statistically significant determinant factors were professional status, working room, and time of personal protective equipment usage.

## 1. Introduction

### 1.1. Background

Acquired human immune deficiency syndrome is disease of the human immune system caused by infection with human immune deficiency virus. Worldwide HIV/AIDS has created enormous challenges on the survival of mankind, and it is transmitted primarily via unprotected sexual intercourse, contaminated blood transmission, hypodermic needles, mucosal (skin) contact with potentially infectious body fluids and from mother to child [1].

The risk of human immunodeficiency virus transmission from patient to health worker is 0.3% and 0.09% following percutaneous and mucocutaneous exposure, respectively. A study conducted in Northern Uganda revealed that 108 (46%) respondents were found to have been exposed to potentially infectious body fluids. Needle stick injuries was the commonest route of exposure, with a prevalence of 27.7%, followed by mucosal exposure (19.1%), contact with broken skin (5.5%) and lastly a cut with sharp objects (5.1%) [2].

Higher circulating viral load in the source patient is also thought to increase the risk of transmission, and evidence of this can include elevated plasma viral load or advanced stage the illness. Many factors can increase the risk of human immunodeficiency virus infection due to occupational exposure in developing countries including Ethiopia. Less stringent safety regulation or standards, unfamiliar practices conditions and limited availability of the personal protective equipment, increased prevalence of injection therapy, unsafe infection prevention practices, performing unfamiliar medical procedures, and limited access to postexposure treatment are the factors that put the healthcare workers at risk for human immunodeficiency virus exposure and other blood-borne pathogens [3].

The risk of occupational exposure to human immunodeficiency virus is most closely related to the activities and duties of the healthcare workers. Occupationally it is transmitted to healthcare workers who are exposed to blood and other potentially infectious injuries and splash exposure to mucosa membrane or nonintact skin. The risk of transmission of human immunodeficiency virus varies with the type and severity of the exposure. Significant risk exposure can defined as percutaneous injury to potential infectious blood, tissue and other body fluids. A health protection agency summary of published reports, looking at human immunodeficiency virus transmission from occupation exposure to human immunodeficiency virus found 22 of 6955 healthcare workers with percutaneous exposure to human immunodeficiency virus become infected, indicating a risk of 1 in 300 or 0.3% [3, 4].

A study conducted in United Kingdom in 4 teaching hospitals showed 175 cases of blood and other body fluids exposure in doctors were reported over the three-year study period. Out of that 81 (46%) occurred in senior doctors and 94 (54.5%) in junior doctors. Junior doctors had higher rate of blood and other body fluids exposure compared to the senior doctors. The most frequent setting for blood and other body fluids exposure among senior doctors was the operating theater/room (59%) [5].

### 1.2. Statement of Problem

A survey of 273 junior doctors at two London teaching hospitals found that 76% had experienced high risk exposure to potentially infective materials during their care [4].

HIV/AIDS is a serious public health problem costing the lives of many people including healthcare workers. Each day thousands of healthcare workers around the world suffer accidental occupational exposures to blood-borne pathogens [6]. It is probably the most serious and causes the highest level of anxiety amongst healthcare workers in many countries including Ethiopia [7].

Ethiopia is no doubt among Sub-Saharan African countries hard-hit by HIV/AIDS. Following the first detection of the virus in 1984, AIDS cases were reported in 1986. The national adult HIV prevalence rate was estimated at 0.2 percent in 1985 increasing to 3.2 percent in 1995 and reduced to 1.4 percent in 2005. A trend analysis carried out for the country from 1982 to 2005 shows a continuous gradual rise of HIV/AIDS prevalence rate until the late 1990s and then a steady decline in the years after 2000. Although this appears encouraging, there is no guarantee that such a trend will continue into the future. According to the 2005 Ethiopian Demographic and Health Survey (EDHS 2005), determinant factors fuelling the prevalence of HIV among the population and wide variations in HIV prevalence exist across regions, the highest in Gambella (6 percent) and Addis Ababa (4.7 percent) [8].

Based on AIDS In Ethiopia 6th reports taken from voluntary counseling and testing service centers, blood banks, and antiretroviral therapy programs, the cumulative number of people living with HIV/AIDS (PLWHA) is about 1.32 million (45% male and 55% female). This results in a prevalence rate of 3.5% (3% among males and 4% among females; 10.5% urban and 1.9% rural areas) for the total estimated population of 73 million. The estimated number of new adult AIDS cases was 137,499. The number of new human immunodeficiency virus infections was 128,922 (353 per day) including 30,338 human immunodeficiency virus-positive births. Females accounted for 53.2% of new infections [9].

A cross-sectional survey conducted in Jimma zone, Oromiya region, southwest Ethiopia, shows that, among the total 254 participants, 174 (68.5%) had ever been exposed to HIV risk conditions. Out of 174 health workers exposed to human immunodeficiency virus risk, 105 (60.3%) sustained needle prick/cut by sharps, 77 (44.3%) to blood, and 68 (39.1%) exposed to patients' body fluid. Perceived causes of exposure were high workload 77 (44.3%), lack of protective barriers 58 (33.3%), and lack of knowledge on standard precautions 31 (17.8%) [10].

Results of the Duke Health and Safety Surveillance System (DHSSS) showed that there were about 2730 blood and other body fluids exposures among healthcare workers. Difference in annual exposure rate was also observed between health professionals of different category and working experience. In study done to estimate the global burden of disease attributable to contaminated sharp injuries, 1000 new human immunodeficiency virus infections have occurred in the year 2000 worldwide among healthcare workers due to their occupational exposure to percutaneous injury [11].

Exposure to body fluids has a potential risk of transmission of blood-borne pathogens that can cause disease such as human immunodeficiency virus and hepatitis to healthcare workers. Healthcare workers are facing a number of unique challenges to stay healthy in the face of generalized HIV/AIDS epidemics. From research findings the estimated risk of HIV/AIDS transmission after injury through needle contamination with human immunodeficiency virus and after mucosa membrane exposure is 0.3% and 0.1%, respectively. Available data from developing countries shows that adherence to the standards precaution and documentation of occupational exposure are suboptimal and the knowledge about the risk factor among the healthcare workers is poor [11–13].

A survey conducted among Serbian healthcare workers depicted that 90% of them carried out some form of intervention with risk of human immunodeficiency virus infection and 70% of them perceived there to be high professional risk of acquiring human immunodeficiency virus infection. Finding from that study showed that, within one year, 59% of healthcare workers had skin contact with patient blood followed by Needle Stick Injuries in 51%, cut from sharp instrument in 38% and contact of eye and other mucosa with patients' blood in 34%. Seventeen percent of Healthcare Workers protected from injury by using appropriate barriers such as glove, glasses, gown, and mask. Nearly 80% of respondents had not been informed about guidelines for protection against human immunodeficiency virus. It was found that perception of professional's risk of acquiring human immunodeficiency virus infection was associated with every day practice, and has higher among healthcare workers who were exposed to patients' blood and other body fluids [13].

Healthcare workers practicing in poor countries like Ethiopia are more exposed to human immunodeficiency virus following occupational exposure [11, 14].

Despite percutaneous occupational exposure still occurs and is under reported. A report published by World Health Organization also estimated that 0.5% of healthcare workers was exposed to human immunodeficiency virus annually, corresponding to an expected 1000 new human immunodeficiency virus infection from occupational exposure [13, 15].

These all poses a high risk of occupational exposure and transmission to healthcare workers in hospitals attending to these patients. Therefore this study will determine the magnitude of occupational exposure among healthcare workers and associated factors in relation to the occupational hazards to human immunodeficiency virus infection.

### 1.3. Significance of the Study

Even though human immunodeficiency virus infection is an epidemic condition all over the world, this study focuses on healthcare workers who are more vulnerable to human immunodeficiency virus exposure due to their occupation. Currently in Ethiopia there is inadequate data about occupational exposure in relation to transmission of blood-borne pathogens among healthcare providers. Healthcare workers in Ethiopia have inadequate knowledge of risky occupational activities, as well as, on the advantage of personal protective equipment and universal precaution during service provision, so that this study will be the source of information for future researchers. Additionally the study helps the healthcare workers to know the severity and prevalence, to minimize its impact, to be safe in their working environment, and remain healthy.

## 2. Literature Review

Worldwide, 4.4% (0.8%–18.5%) of human immunodeficiency virus infection among healthcare workers may be attributable to occupational injuries. More than 90% of the infection occurred in low income country, most of which could have been prevented [15].

Worldwide occupational exposure accounts for 2.5% of human immunodeficiency virus cases among healthcare workers. Each year as consequence of occupational exposure, estimated up to 1000 human immunodeficiency virus infection occur among healthcare workers. According to World Health Organization estimation of 3 million percutaneous exposures occurs annually, among which 35 million healthcare workers globally, over 90% occurring in resource constricted countries [15, 16].

In the study conducted in Ethiopia, in Tigray region showed that, out of the total 618 healthcare workers interviewed about occupational exposure in the past three months, 106 (17.2%) had experienced needle stick injury, 348 (56.3%) had contact of blood and body fluid with their skin, and 154 (24.9%) reported exposure to their mucosa membrane. Working in the delivery room (80.4%) and gynecological wards (75%) had higher risk of exposure [17].

Study done on accidental occupational exposure of healthcare workers in Kenya Rift Valley Provincial Hospital shows that 19% of healthcare workers reported having percutaneous injury, 7.2% splash to mucosa membrane, and 25% exposure to blood and other body fluids in the past 12 months. Higher rate of percutaneous injuries was observed among nurses (50%) during stitching (30%) and in obstetric and gynecologic department (22%) healthcare workers aged below 40 [16].

The risk of healthcare workers to acquire human immunodeficiency virus or blood borne pathogens after an occupational exposure depends on multiple factors like high prevalence of the infection in the specific population, frequency of exposure (activities capable of transmitting the infection agent), nature and efficiency of transmission of exposure (percutaneous injury has increased risk of transmission compared with exposure to mucosa membrane or skin), high viral load, or patients with advanced illness [18].

A research done on occupational risk of human immunodeficiency virus information among western healthcare professional posted in AIDS endemic areas showed that out of 99 Dutch medics working 65% reported percutaneous exposure during an average stay of 21 months. The mean number of injuries was lower among physicians (2.0 versus 3.9 per year) and higher among nurses [19].

Another study done to know the risk of healthcare workers in developing countries found that healthcare workers in developing countries are at the serious risk of infection from blood-borne pathogens particularly human immunodeficiency virus, hepatitis B virus and hepatitis C virus because on the high prevalence of such pathogens in many regions of world [20]. It was also pointed out that, despite the high prevalence of blood borne pathogens in many developing countries, documentation of infection caused by occupational exposure is inadequate [20–22].

Another study done on specific groups of health professional indicates that occupational exposure is real risk and is attributable to some work related factors [22, 23]. The study done on nurse showed that the percentage of nurses experiencing needle stick injury during their professions time was 79.7%. The rate of needle stick injury among the study subjects was high in subjects with age less than 24 and less than 4 years of nursing experience, working in the surgical intensive care units, and working for more than 8 hrs per day [23].

Some other studies also indicated that injuries were more frequent during extended work compared with none extended and the extent of occupational exposure among healthcare workers was significant. Decreasing the work load of some specific healthcare workers was indicated as one measure of decreasing the occurrence of exposure [24–26].

In survey conducted among emergency medical residents in United States of America 56.1% reported at least one exposure to blood during their training. The frequency of self-reported exposure increased with advancing level of training. They were frequently exposed to blood, most commonly due to puncture of sharp objects but rate of exposure reporting was low, which may compromise appropriate post exposure counseling and prophylaxis [27].

Study conducted among primarily healthcare workers in Ghana showed that 21% of staff perceived that they were not at risk of exposure to blood-borne virus although potentially exposed. Educationally intervention was found to be effective to increase the knowledge of healthcare workers on occupational exposure [28]. Another study done in South Africa about knowledge of physicians on occupational risk of human immunodeficiency virus infection dedicated that 83.3% of the respondents did not appreciate the true occupational risk of human immunodeficiency virus infection and 31% did not know that needle stick injury is the commonest mode of occupational acquisition [29].

**Figure 1:**
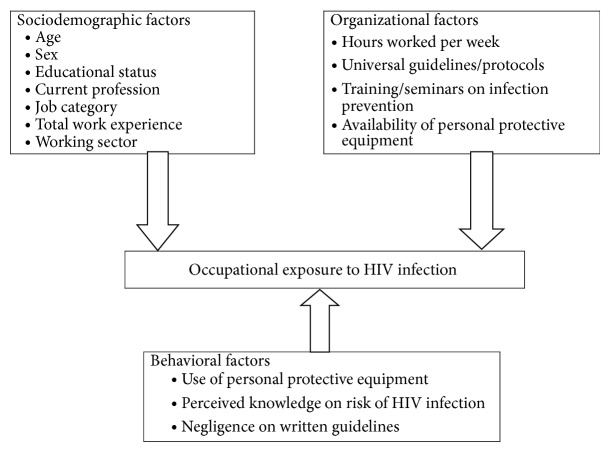
Conceptual framework and variable specification for the study in Debre Berhan town, Ethiopia, 2014.

Even though the degree of exposure varies from time to time or from health institution to health institution, generally healthcare workers occupational exposure to human immunodeficiency virus infection is prevalent. It was also pointed out that, despite the high prevalence of blood-borne pathogens in many developing countries, documentation of infection caused by occupational exposure is inadequate. The factors that are attributable to this are categorized as sociodemographic, work environment or organizational factors, and behavioral factors. Among those the most common determinant factors are year of working experience, total working hours per week, number of patient, and perceived knowledge on risk of human immunodeficiency virus infection. The association of these factors is depicted by conceptual frame work in Figure 1, which is developed after reviewing the literatures.

## 3. Objective

### 3.1. General Objective

The purpose of this paper is to assess prevalence and determinants of occupational exposure to human immunodeficiency virus infection among healthcare workers in selected health institutions in Debre Berhan town, North Shoa zone, Amhara region, Ethiopia.

### 3.2. Specific Objective

The specific objects are as follows:

- To determine the prevalence of occupational exposure to human immunodeficiency virus infection among healthcare workers.
- To identify determinant factors associated with the risk of occupational exposure to human immunodeficiency virus infection among healthcare workers.

## 4. Methods and Materials

### 4.1. Study Design

A descriptive cross-sectional institution based study was conducted to assess the prevalence and determinant factors of occupational exposure to human immunodeficiency virus infection among healthcare workers in selected health institution in Debre Berhan town, North Shoa zone, Amhara region, Ethiopia.

### 4.2. Study Area and Period

Debre Berhan town is one of the administrative towns in Ethiopia, Amhara region, which is the capital city of North Shoa zone and it is located 130 kilometers from Addis Ababa to the North East. The town has 9 kebeles with total population of 94,829; out of this 50.8% are females. In the town there are about 22 health institutions, two hospitals (one private and one referral governmental hospital), 3 health centers, and 17 private clinics. The study was carried out in the selected four health institutions, which are two hospitals and two health centers from January to June 2014.

### 4.3. Source Population

All health care workers were employed in all health institutions of Debre Berhan town, North Shoa zone, Amhara region, Ethiopia.

### 4.4. Study Population

All health care workers were employed in Debre Berhan Referral Hospital, Ayu General Hospital, Kebele 04 Health Center, and Tebase Health Center.

### 4.5. Inclusion and Exclusion Criteria

#### 4.5.1. Inclusion Criteria

All healthcare workers working in the selected health centers and hospitals who have a potential to be exposed to human immunodeficiency virus in their day to day professional activities such as nurses, physicians, laboratory clinician, midwifes, and health officers.

#### 4.5.2. Exclusion Criteria

Administrative and technical workers in which their day to day activities do not make them at risk of acquiring or being exposed to human immunodeficiency virus infection due to their working place; for example, managers, secretaries, porters, finance, security staffs, and pharmacists were excluded. Additionally students who were temporarily assigned for training purpose also excluded.

### 4.6. Sample Size Determination

The actual sample size for the study was determined using the formula for single population proportion. To determine the initial sample size the following assumption was made: assuming 5% marginal error (*d*), 95% confidence level (alpha = 0.05), and the proportion or prevalence of healthcare workers occupational exposure to human immunodeficiency virus infection to be 50%. So based on the above information the total sample size was calculated by using the following:

(1) ni=(Z2α)2P1−pd2,

where *n*_*i*_ = initial sample size from finite population, *Z* = the standard score (critical value) corresponding to 95% confidence level, *P* = the proportion of healthcare workers experiencing occupational exposure which is taken 50%, 1 − *p* = is proportion of negative character, and *d* = marginal error which is taken to be 5%:

(2) ni=z2α2p1−pd2,ni=1.9620.51−0.50.052,ni=384.

Since sampling is made from a finite population (*N* = 476) which is less than 10,000, it needs the finite population correction. Therefore

(3) nf=ni(1+ni)/N, where  N=size  of  the  source  population=3841+384/476=212.6≈213.

By considering 10% nonresponse rate, the total final sample size will be

(4) nf=ni+contingencyni,nf=213+213×10%,nf=213+21.3=234  health  care  workers.

### 4.7. Sampling Procedure

From total of 22 health institution found in Debre Berhan town, both governmental and private health institutions, only four (Debre Berhan Referral Hospital, Ayu General Hospital, Kebele 04, and Tebase Health Centers) were selected purposefully, because the remaining 17 clinics and health centers were not sufficient to generalize for the whole healthcare workers in the town due to provision of limited healthcare service and a small number of staffs were employed. To determine the total participants from each selected health institution stratified sampling technique was utilized.

### 4.8. Variables

*Dependent Variables*

- Occupational exposure of healthcare workers to human immunodeficiency virus infection.

*Independent Variables*

- Sociodemographic factors.
- Organizational factors.
- Behavioral factors.

### 4.9. Data Collection

Quantitative and qualitative data were collected by using semistructured interviewer administered questionnaire. The questionnaire has three parts; part one: sociodemographic characteristics of healthcare workers, part two: organizational and behavioral factors, and part three: occupational exposure to human immunodeficiency virus infection. The questionnaire was prepared in English, later translated in to local language Amharic for appropriate and easiness during interviewing. The Amharic version was again cross-checked for its consistency of meaning with the English version. Translation of questionnaire was done by language experts in both cases. To collect the data each respondent was invited to a private room for face-to-face interview with data collectors.

### 4.10. Data Quality Control

To assure quality of the data, properly designed data collection tool was prepared and pretested and training was given to data collectors. Additionally, on each data collection day, the collected data was reviewed and checked for its completeness by principal investigator and appropriate design and sampling procedures were applied. Moreover, the exclusion criteria were considered.

### 4.11. Data Processing and Analysis

After checking collected data visually for completeness, each frequency distribution of variables was worked out using hand tally rather than entering and analyzing by using Epi Info or Epi data or SPSS (Statistical Package for Social Science). The frequency distribution of dependent and independent variables was organized by frequency table, graph, and chart. Chi-square and odds ratio were used to determine the associations between the selected variables. *P* value was also calculated to identify possible statistically significant risk factors.

### 4.12. Ethical Consideration

Ethical clearance was obtained from research committee of Debre Berhan University Institute of Medicine and Health Science. The data collectors introduced themselves, explained the objectives and benefits of the study, informed the respondents that they can participate or refuse at any time they want and they have a chance to ask anything about the study. Data was collected anonymously after obtaining verbal consent from each respondent by assuring confidentiality throughout the study period.

### 4.13. Pretest

Pretest was conducted on healthcare workers working in health institutions other than selected for the study before the actual data collection. Modification on logical sequence, simplicity, and clarity of questionnaire was done.

## 5. Results

### 5.1. Sociodemographic Characteristics

A total of 234 respondents were included in the study at Debre Berhan Referral Hospital, Ayu General Hospital, Tebase Health Center, and Kebele 04 Health Center with a response rate of 90.2%. Among the total respondents, 187 (88.6%) had been encountered risky occupational exposure to human immunodeficiency virus infection.

As shown in Table 1, 123 (58.3%) of the study participants were male, 82 (38.9%) were within the age range of 25–29 years (mean age = 30), and 114 (54%) of them were diploma graduate. In addition about 119 (56.4%) of the study participants were nurses, 157 (74.4%) of the participants had working experience <10 years, and 120 (56.9%) were working >40 hours/week. Majority 194 (81.1%) of them work currently in Debre Berhan Referral Hospital.

### 5.2. Existing Knowledge, Perception, and Practice (Behavioral Factors)

Of all the participants of the study 155 (73.5%) knew what occupational exposure to HIV/AIDS mean. Eighty-seven point seven percent (*n* = 185) of the respondents perceived that being a healthcare worker can expose them to HIV/AIDS due to daily care provision.

Majority (67.8%) of the respondents knew that needle stick injury, cutting injury, glove breakage skin contact with blood semen, amniotic, and other body fluids are the common occupational risk to acquire human immunodeficiency virus infection. 174 (82.5%) of respondents perceived that they were at risk of exposure to human immunodeficiency virus due to their profession, and out of them 66 (37.9%) and 108 (62.1%) were classified their risk status as high and low risk level, respectively.

About 37 (17.5%) of respondents perceived they were not at risk of exposure to human immunodeficiency virus due to their profession because they take care of themselves while they provide care for their patient. Out of 174 respondents perceived that they were at risk due to their profession, 145 (83.3%) of them knew the correct measure that prevents occupational risk exposure to human immunodeficiency virus.

Out of the total study participants 123 (58.3%) were reported that training and other seminars on universal infection prevention were given in their health institution; among them only 94 (76.4%) were trained.

Eighty-seven point seven percent of the study participants reported that universal precautions and guidelines about infection prevention had been posted in their health institution; among them only 130 (70.3%) were followed the posted guide lines.

Majority (92.4%) of the respondents knew all the types of personal protective equipment. Out of the total respondents, nearly all (95.7%) perceived that personal protective equipment can prevent from occupational risk to human immunodeficiency virus acquiring.

Four point three percent of the respondents perceived that personal protective equipment cannot prevent occupational risk as well as they failed to use this equipment. Perceived reason for not using personal protective equipment of healthcare workers was shown in Table 2.

**Figure 2:**
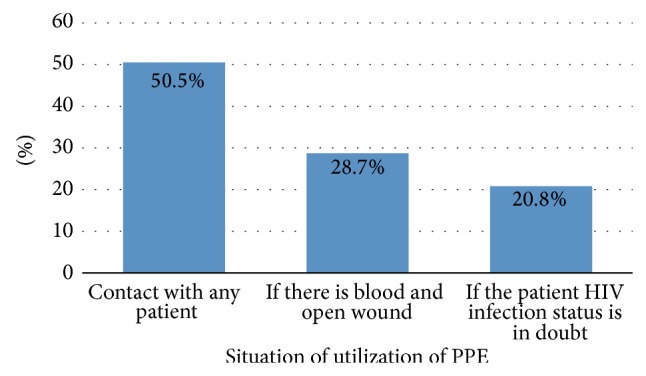
Situation of utilization of personal protective equipment by healthcare workers in Debre Berhan town, 2014.

As shown in Figure 2, half of the respondents 111 (50.5%) were used personal protective equipment while contact any patient.

**Figure 3:**
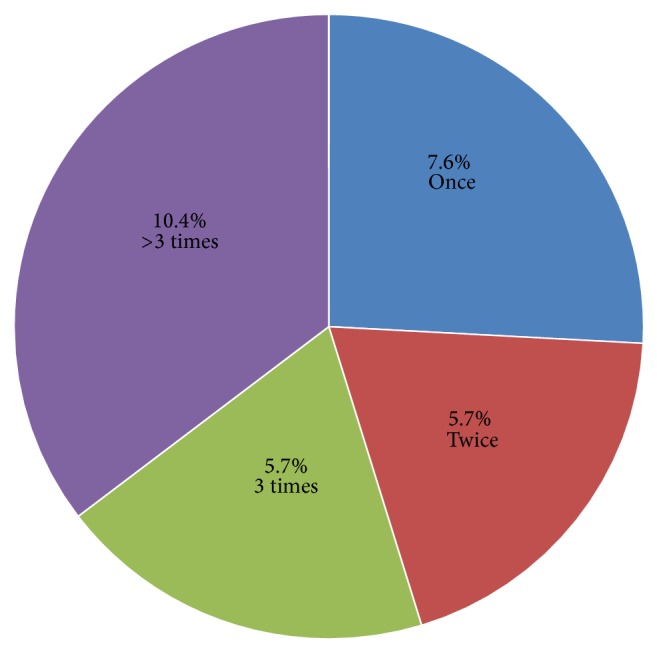
Frequency of use of personal protective equipment during emergency care provision by healthcare workers in the last 12 months in Debre Berhan town, 2014.

Out of the total respondents, majority, 149 (70.6%), were used personal protective equipment during emergency care provision in the last 12 months. As depicted in Figure 3, 10.4% of health care workers use personal protective equipment >3 times during emergency care provision in the last 12 months.

**Figure 4:**
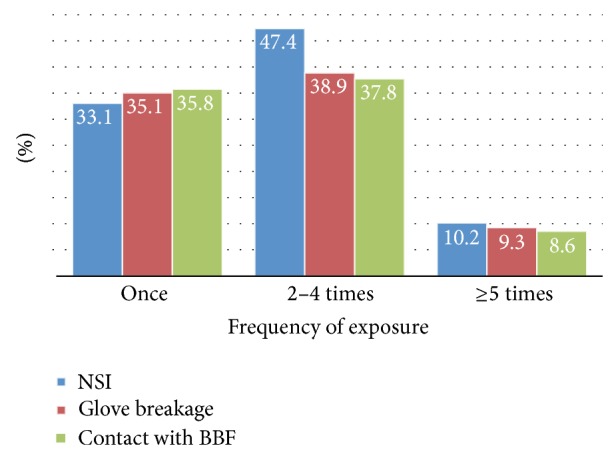
Needle stick injury (NSI), glove breakage, and contact with potentially infectious blood and other body fluids versus frequency of exposure among healthcare workers in Debre Berhan town, 2014.

From Figure 4, 59 (31.5%) of the respondents had been exposed to needle stick injury, among them 33.9%, 47.4%, and 10.2% were in the range of 1, 2–4, and ≥5, respectively.

**Figure 5:**
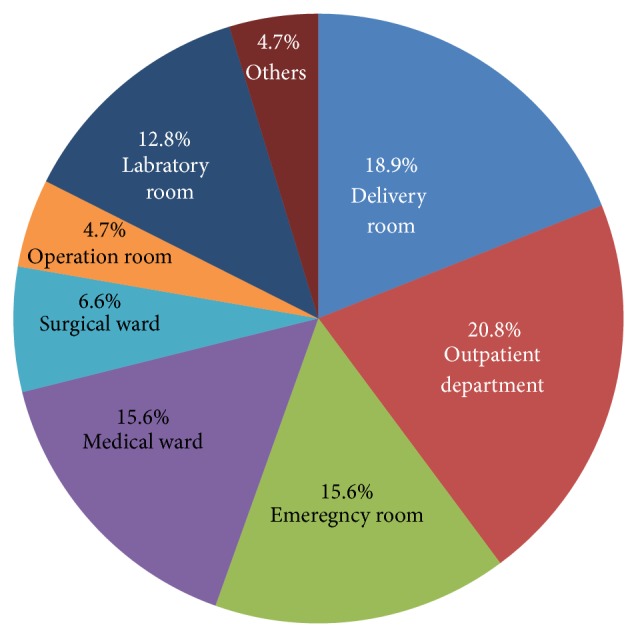
The percentage of working room of healthcare workers of the last 12 months in the selected health institutions, 2014.

As shown in Figure 5, 44 (20.8%) and 10 (4.7%) were working in outpatient department (OPD) and operation room, respectively.

**Figure 6:**
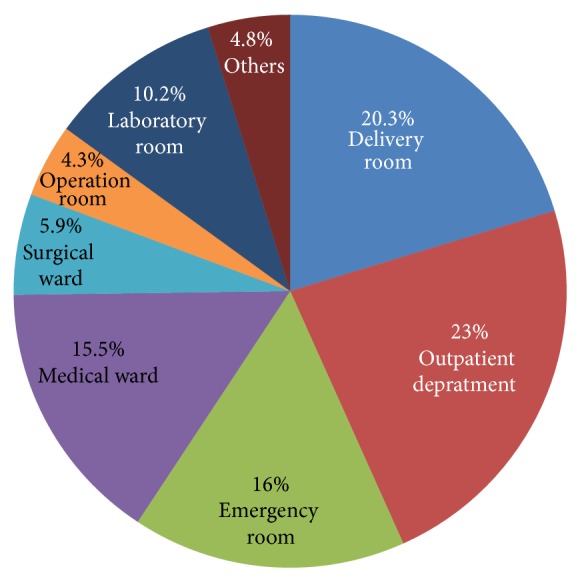
The percentage of healthcare workers in different working unit who had been experiencing needle stick injury, contact with potentially infectious blood and other body fluids, and glove breakage in the past 12 months in selected health institutions, 2014.

Among those healthcare workers who are working in different unit most of them had been experiencing needle stick injury, contact with potentially infectious blood and other body fluids and glove breakage in past 12 months. As illustrated in Figure 6, 23% and 4.3% of health care workers had been experiencing needle stick injury, contact to potentially infectious blood and other body fluids, and glove breakage in the past 12 months in outpatient department and operation room, respectively.

Based on Table 3, healthcare workers who had extended working hours (>40 hrs/week) and work experience (≥50 years) were 1.2 and 1.4 times higher to be exposed to needle stick injury, contact with blood and other body fluids, and glove breakage, respectively. Correspondingly, those personal protective equipment users were 1.6 times higher to be exposed than nonusers. This can be attributable to the time (condition) of personal protective equipment usage and quality of personal protective equipment. However, healthcare workers who were trained about infection prevention were 0.78 times less likely to be exposed than those who were not trained about infection prevention. Additionally, female healthcare workers were less likely to be exposed than males.

### 5.3. Results of Chi Square

(i) * Association between professional status and exposure*
  (5) $$\begin{array}{c} X^{2}=13.4 \end{array}$$
  (13.4 corresponds to *P* value of <0.02).
 Therefore, there is a relationship between professional status and occupational exposure status of healthcare workers.
(ii) * Association between working room and exposure *
  (6) $$\begin{array}{c} X^{2}=14.4\;\left(P<0.05\right). \end{array}$$
  Therefore, there is a relationship between working room and occupational exposure status of healthcare workers.
(iii) * Association between time of personal protective equipment usage and exposure*
  (7) $$\begin{array}{c} X^{2}=15.1\;\left(P<0.001\right). \end{array}$$
  Therefore, there is a relationship between time (condition) of personal protective equipment usage and occupational exposure status of healthcare workers.

But educational status had not shown association with occupational exposure status of healthcare workers.

## 6. Discussion

In this study female healthcare workers were less likely to be exposed than males. This finding was consistent to the study finding done in Northern Uganda [2].

The results of this study shows that nearly all (88.6%) of the healthcare workers who participated in the study had experienced risky occupational exposure during their professional activities in the last one year. This found to be higher than the findings of other studies conducted in London teaching hospitals (76%) [4], United States of America emergency medical hospital (56.1%) [27], Kenya rift valley provincial hospital (51%) [16], and Jimma zone, Oromiya region, southwest Ethiopia (68.5%) [10]. This difference might be due to small sample size of this study, variation in the study area, and economic development variation of nations. But this finding was less than the study done in Serbia (98.4%) [13].

Regarding the mode of exposure to human immunodeficiency virus this study revealed that 59 (31.6%) of the healthcare workers experienced needle stick injury, 54 (28.9%) glove breakage, and 106 (56.6%) of them experienced contact with potentially infectious body fluids. This result was nearly similar with the study conducted in Northern Uganda [2], which was 27.7% of the healthcare workers experienced needle stick injury. But this finding was less than the study done in Serbia [13], which was 89% had needle stick injury, 59% skin contact with potential infections blood and fluids and findings of the study done Jimma zone, Oromiya region, southwest Ethiopia [10], which was 105 (60.3%) sustained needle prick/cut by sharps, 77 (44.3%) to blood, and 68 (39.1%) exposed to patients' body fluid. Moreover, it was higher than the results in Kenya [16] which was 19% had needle stick injury, 25% had contact with blood and other body fluids in the past one year and results of the study conducted in Ethiopia, Tigray region [17] which was 17.2% had needle stick injury, 56.3% contact with blood and body fluid in the past 12 months. This discrepancy could be due to difference in background of healthcare workers, difference in the sample size, and the concern of employing organization.

Likewise, in this study most of the respondents were exposed to risky condition 2–4 times. This finding was similar with finding of the study done in Northern Uganda [2].

In addition, this study shows that working in outpatient department (23%), followed by delivery room (20.3%), and emergency room (16%) had high risk of exposure, which was different from the results of the study done in Tigray region [17] that was 80% working in delivery room and 75% in gynecological wards. This discrepancy might be due to difference in the service delivered by facilities.

The result of this study showed that 82.5% of the respondents perceived that they were at risk of exposure to human immune deficiency virus infection due to their profession. This finding was almost similar to result of healthcare workers in Ghana (79%) [28]. This might be due to the effect of globalization, global burden of the HIV/AIDS, and internet technology that was helpful to update themselves.

This study show that majority (67.8%) of the healthcare workers identify the commonest occupational risk to human immunodeficiency virus infection. This finding was completely contradictory to the report from South Africa [29] which was 83.3% of the healthcare workers did not identify. This discrepancy might be due to lack of training, variation in organization safety policies and procedures, and less exposure to the occupational hazards.

Nearly all the study participants were informed about the universal precautions and guidelines about infection prevention. In contrary to this finding, the study conducted in Serbia [13] discovered that 80% of the health care workers had not been informed. This might be due to variation in training to healthcare workers on universal precaution to decrease the rate of occupational exposure.

In this study 95.7% of the respondent was used personal protective equipment, only 23% of them were protected from injury. This was almost similar to Serbian healthcare workers that only 17% had been protected [13]. This does not mean that using personal protective equipment can expose healthcare workers to injury but it is the fail to use them at appropriate time and condition and problem in the quality of the personal protective equipment.

## 7. Conclusion

This study was likely to be the actual reflection of the occupational exposure to human immunodeficiency virus infection of health workers in selected four health institutions of Debre Berhan town. Even though most of healthcare workers were informed as well as being familiar to universal standards and guidelines, this study revealed that majority (88.6%) of healthcare workers had experienced the risky occupational exposure during the past 12 months. A number of determinant factors which were potential acquisition of occupational hazards were identified. The statistically significant determinant factors were professional status, working room, and time (condition) of personal protective equipment usage.

## 8. Recommendation

The North Shoa Zone Health Bureau believes it is better to conduct training and seminars about infection prevention to create awareness among healthcare workers. Furthermore reducing the working hour was also advantageous to healthcare workers.

Debre Berhan Health Institution and Healthcare Workers believe it is better to increase the availability of personal protective equipment, practicing organizational safety policies, and procedures as well as practicing the appropriate time and condition in using those personal protective equipment that decreases the chance of exposure to risky occupational hazards.

## 9. Strength and Limitation of the Study

The strengths of this study include high response rate and the inclusive nature of this research as individuals could participate regardless of their profession. Additionally a reasonable sample size and culturally adapted questionnaires were used. Since it was the first study in type in the study area, it will provide basic information for those who are interested.

However an important limitation of this study was that the study relies on self-report rather than having record review of healthcare workers. Due to cross-sectional nature of the study causal relationships between the risk factors and occupational exposure to human immunodeficiency virus infection could not be assumed. Furthermore, the data was analyzed manually and chi-square model which was a weak measure of association was utilized.

## Figures and Tables

**Table 1:** Sociodemographic characteristics of the healthcare workers in Debre Berhan town, 2014.

Variables	Category	Frequency	Percent (%)
Age (in years)	20–24	41	19.4
	25–29	82	38.9
	30–34	36	17.1
	35–39	25	11.8
	40–44	12	5.7
	45–49	8	3.8
	50–54	2	0.9
	≥55	5	2.4

Sex	Male	123	58.3
	Female	88	41.7

Minimum qualification obtained	Diploma	114	54
	Bachelor Degree	88	41.7
	Masters Degree	8	3.8
	Other higher level	1	0.5

Profession	Specialist	5	2.4
	General practitioner	19	9
	Nurse	119	56.4
	Health officer	26	12.3
	Mid-wives	20	9.5
	Laboratory technician	22	10.4

Working sector	Debre Berhan Referral Hospital	194	81.1
	Ayu General Hospital	18	8.5
	Kebele 04 Health Center	11	5.2
	Tebase Health Center	11	5.2

Total work experience (year)	<10	157	74.4
	≥10	54	25.6

Working hour/week	≤40	91	43.1
	>40	120	56.9

**Table 2:** Perceived reason for not using personal protective equipment of healthcare workers in Debre Berhan town, 2014.

Reason for not using PPE	Frequency	Percent (%)
Doubt in their prevention capacity	2	22.2
Forgetting	2	22.2
Allergy	0	0
Not available	5	55.6

Total	9	100

**Table 3:** Association of variables with risky occupational exposure to needle stick injury, glove breakage, and contact potentially infectious blood and other body fluids.

Variables		Exposed	Nonexposed	Odds ratio
Working hour/w.k.	>40	108	12	1.2
	≤40	80	11	

Total work experience (year)	≤10	52	5	1.4
	>10	137	18	

Training about infection prevention	Yes	82	12	0.78
	No	105	12	

Personal Protective Equipment (PPE)	Yes	175	27	1.6
	No	7	2	

Sex	F	45	3	0.3
	M	54	1	
